# Assessing Psychodynamic Conflicts and Level of Personality Functioning in Women Diagnosed With Vaginismus and Dyspareunia

**DOI:** 10.3389/fpsyg.2021.687369

**Published:** 2021-06-24

**Authors:** Thula U. Koops, Christian Wiessner, Johannes C. Ehrenthal, Peer Briken

**Affiliations:** ^1^Institute for Sex Research, Sexual Medicine, and Forensic Psychiatry, University Medical Centre Hamburg-Eppendorf, Hamburg, Germany; ^2^Institute of Medical Biometry and Epidemiology, University Medical Centre Hamburg-Eppendorf, Hamburg, Germany; ^3^Department of Psychology, University of Cologne, Cologne, Germany

**Keywords:** female sexual difficulties, vaginismus, dyspareunia, genito-pelvic pain/penetration disorder, operationalized psychodynamic diagnosis (OPD)

## Abstract

Knowledge on etiological and risk factors of genito-pelvic pain/penetration disorder, formerly classified as dyspareunia and vaginismus, is limited. The Operationalized Psychodynamic Diagnosis (OPD) system offers a valuable basis for developmental considerations, and has not yet been used to research sexual pain difficulties in women. We conducted an exploratory pilot study of psychodynamic motivational conflicts and level of personality functioning as defined by the OPD system by means of an anonymous online survey among 24 women who had been diagnosed with dyspareunia or vaginismus. We matched them with 24 healthy controls and compared groups using paired-samples *t*-tests and Wilcoxon tests. Effect sizes were calculated using Pearson's *r*. Large effect sizes were found for mean or median differences of several OPD Structure Questionnaire (OPD-SQ) scales (self-reflection, *p* = 0.002/*r* = 0.59; affect differentiation, *p* = 0.007/*r* = 0.53; self-perception, *p* = 0.002/*r* = 0.58; impulse control, *p* = 0.007/*r* = 0.53; self-worth regulation, *p* = 0.008/*r* = 0.52; self-regulation, *p* = 0.004/*r* = 0.56; experiencing affect, *p* = 0.009/*r* = 0.53; bodily self, *p* = 0.008/*r* = 0.54; OPD-SQ total score, *p* = 0.007/*r* = 0.52; internal communication, *p* = 0.001/*r* = 0.63) and OPD Conflict Questionnaire (OPD-CQ) scales (guilt conflict active, *p* = 0.004/*r* = 0.60; Oedipal conflict passive, *p* = 0.009/*r* = 0.51; individuation versus dependency conflict active, *p* = 0.01/*r* = 0.52; guilt conflict passive, *p* < 0.001/*r* = 0.70; self-worth conflict passive, *p* = 0.001/*r* = 0.70; passive mode, *p* < 0.001/*r* = 0.68). The problems with personality functioning and more pronounced types of conflicts participants displayed suggest proneness for self-invalidation, internalization and restricted self-perception.

## Introduction

Vagi1nismus and dyspareunia have long been the two available diagnoses to characterize women's sexual difficulties associated with pain which are not due to a known physiological condition (World Health Organisation, 1994; American Psychiatric Association, 2000). Concerning their diagnostic validity and differentiation, a considerable amount of uncertainty and disagreement had been expressed both by clinicians and researchers over the decades (see for example Meana et al., 1997; Reissing et al., 1999, 2004; Ohkawa, 2001; Binik et al., 2002). The latest revision of the Diagnostic and Statistical Manual of Mental Disorders (DSM-5; American Psychiatric Association, 2013) and the International Classification of Diseases (ICD-11; World Health Organisation, 2019) therefore involved a reclassification of vaginismus and dyspareunia as one diagnostic category (with different conceptualizations), named *genito-pelvic pain/penetration disorder* (DSM-5) and *sexual pain-penetration disorder* (ICD-11). However, a lack of clarity about the phenomena of painful or infeasible intercourse in women remains, as reflected in the DSM-5 manual's statement that “the development and course of genito-pelvic pain/penetration disorder is unclear” (American Psychiatric Association, 2013). Moreover, there is a noticeable absence of psychodynamic considerations in the literature covering their etiology and risk factors, despite the general recognition of psychodynamic diagnostics and therapy as essential elements of psychotherapeutic treatments of women's sexual difficulties (Leiblum and Wiegel, 2002; Bitzer and Brandenburg, 2009) and the usefulness of psychodynamic concepts for developmental formulations.

The Operationalized Psychodynamic Diagnosis (OPD) system provides a framework for standardized diagnosis, treatment planning, and process evaluation on a psychodynamic basis. Created in 1992, it has been since reissued in a revised and extended version (OPD-2; Arbeitskreis OPD, 2006) and tested for its validity and reliability (e.g., Cierpka et al., 2007; Schneider et al., 2008; Benecke et al., 2009; Zimmermann et al., 2010; Doering et al., 2014). The OPD system's manual has been translated into several languages like Chinese, English, Hungarian, Italian, Portuguese, Spanish, and Russian (Vicente et al., 2012; Dinger et al., 2014). OPD diagnoses are set out to support clinical purposes by providing guidelines for treatment and structure for therapists in training, as well as research by delivering a tool for studies of psychodynamic concepts and processes. It further improves communication about psychodynamic concepts, which is beneficial to both clinical and scientific work. For a recent overview see Ehrenthal and Benecke (2019).

The study described here was based on the OPD-2 manual which includes the original basic OPD concepts plus adaptations in accordance with the experiences made with the first version, for example a stronger orientation toward therapeutic process diagnostics or patients' resources (cf. Cierpka and Strauß, 2006). An OPD-2 diagnosis consists of an evaluation on five axes: (I) experience of illness and prerequisites for treatment; (II) interpersonal relations; (III) conflicts; (IV) structure; and (V) mental and psychosomatic disorders as diagnosed in the established descriptive-phenomenological diagnostic systems (ICD-11; DSM-5). Each axis requires ratings of its respective components which are then brought together in a global assessment. The system conceptualizes repetitive maladaptive interpersonal patterns as a phenomenon that is fueled by either intrapsychic psychodynamic conflicts (i.e., by a combination of wishes and related defenses), by deficits in personality functioning, referred to in the OPD system as “structure” (i.e., deficits in developmentally acquired basic psychological capacities), or a combination of both. OPD-2 describes seven distinct topics for conflictual development: closeness, agency, care, self-worth, guilt, roles, and identity. The main conflict's mode of processing – meaning the predominant style of how the conflict is attempted to be managed Gisch et al. (2020) – is rated as active (i.e., exaggerated autonomy, need for control, care for others, self-praise, rejection of responsibility, placing oneself in the middle of attention, and identity adoption), passive (i.e., exaggerated need for closeness, giving up control, need for care, self-devaluation, taking over responsibility, hiding from attention, and obfuscating identities), or a mixture of both. Furthermore, it defines eight structural functions with three subdimensions each in the areas of perception/cognition, regulation, communication, and attachment, both with regard to the self as well as others. With axis I being predominantly treatment-related and axis II rather describing maladaptive repetitive patterns, but not their etiology (OPD Task Force, 2008), axis III and IV were considered most suitable for our study purpose.

Originally, an OPD evaluation was based on a 1 to 2 h interview followed by a rating by the clinician or an external observer. But given the time and training such expert interviews require, an increasing demand for time-economic measures led to the development of screening instruments for the structure axis (OPD-SQ; Ehrenthal et al., 2012, 2015; and its short version, OPD-SQS) and the conflict axis (OPD-CQ; Benecke et al., 2018).

So far, the OPD system has not been applied in research on women with persistent or recurrent experiences of painful or infeasible intercourse. This exploratory pilot study is a first step to gain insight into psychodynamic conflicts and the level of personality functioning as defined by OPD-2 among this group in comparison with a control group.

## Materials and Methods

### Design

In our clinical sample we included women who were at least 18 years of age, fluent in the German language, and had been diagnosed with dyspareunia or vaginismus (given that DSM-IV diagnostic categories still applied at the time) by a psychotherapist from our outpatient clinic, or a gynecologist.

Participants of a previous interview study were contacted and invited to take part in the survey. Additionally, women who in the past had sought counseling at our outpatient clinic, and who had given permission to be approached for research purposes, were contacted by the first author. All were provided with a link to anonymously access an online survey via e-mail. Control sample data stemmed from previous OPD validation studies (see *Analysis* section).

Due to stagnation of participation over the course of the data collection period, we introduced a compensation in the form of a 30€ Amazon voucher for following participation, funded by the Institute for Sex Research, Sexual Medicine, and Forensic Psychiatry. Therefore, participants who completed the survey could opt to be redirected to an independent page and register for the voucher with their e-mail address. The access link was distributed via e-mail. Eight participants received compensation.

Ethical approval of our study was granted by the ethical review board of the Hamburg Chamber of Psychotherapists.

### Clinical Sample

Twenty-nine women completed the online survey. With *genito-pelvic pain/penetration disorder* being the diagnosis available for women's sexual pain complaints at the time of data collection, DSM-5 diagnostic criteria were screened in the survey. Datasets of five participants had to be excluded from analysis as DSM-5 diagnostic criteria for were not fulfilled (no complaints in the last 12 months at all; no or only slight perceived distress because of complaints). Table 1 gives an overview of sample characteristics.

**Table 1:**
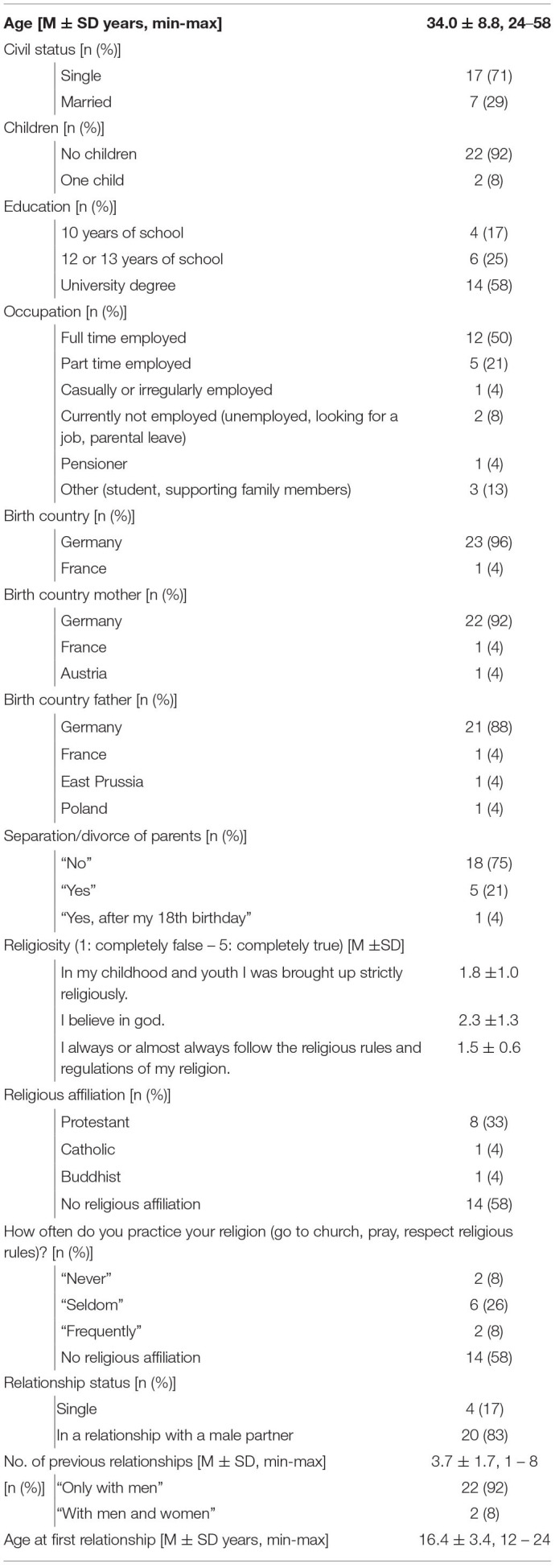
Sample characteristics clinical sample (*n* = 24).

*Percentages rounded; M, mean; SD, standard deviation*.

### Materials

The following questionnaires were used to assess psychodynamic conflicts and the level of psychic structural integration according to the OPD-2 system among participants from the clinical sample as well as further constructs for sample description. Additional data regarding sociodemographic characteristics, religiosity, and relationships were collected.

#### Sample Description – Psychological Problems and Quality of Life

SCL-K-9 is the short version of the Symptom Checklist (SCL-90-R; Franke and Stäcker, 1995). Its nine items measure subjective distress about different psychological symptoms in the past week on a 5-point Likert scale from 0 (“Not at all”) to 4 (“Extremely”). Prinz et al. (2013) determined an internal consistency of α = 0.84 for SCL-K-9. Norm values for the German population have been determined by Petrowski et al. (2019).

WHOQOL-BREF is the short version of the WHOQOL-100 (WHOQOL Group, 1994) and assesses quality of life over the last two weeks in four domains: physical health, psychological health, social relations, and environment. It consists of 26 items with a 5-point Likert scale format inquiring “how much”, “how completely”, “how often”, “how good”, or “how satisfied” respondents felt. Scores can be reported as absolute values or transformed into a percentage scale, with 100% referring to the highest quality of life state. Its good psychometric properties have been confirmed in studies from a wide range of countries (see Skevington et al., 2004).

#### Sample Description – Sexual Difficulties

The Female Sexual Function Index (FSFI) (Rosen et al., 2000) queries self-reported sexual difficulties in the past four weeks in six domains: sexual desire, arousal, lubrication, orgasm, satisfaction, and pain. Items on sexual desire and satisfaction are rated on a 5-point Likert scale from 1 (“almost never/never” or “very unsatisfied”) to 5 (“almost always/always” or “very satisfied”); the remaining items contain the extra option 0 (“no sexual activity”, with “sexual activity” being described as including mutual caressing, foreplay, sexual intercourse, or masturbation, or “no sexual intercourse” for pain and vaginismus). Lower scores refer to more pronounced sexual complaints. As the number of items varies between scales, sum values are adjusted so that a maximum score of 6 can be reached on each scale. The FSFI usually consists of 19 items; in accordance with Peixoto and Nobre (2015) we added an items assessing experiences of vaginismus. The FSFI is an internationally established and widely used instrument (for cross-validation and commonly used clinical cutoff scores see Wiegel et al., 2005; for a review of sexual difficulties and pain prevalence in women worldwide based on the FSFI see Koops and Briken, 2018).

The screening instrument for DSM-5 sexual dysfunctions refers to the last 12 months and includes three domains for women (lack of sexual interest, problems with orgasm, pain during sexual intercourse), on a 5-point Likert scale from “never” to “always/nearly always”. For existing sexual complaints, it assesses the degree of distress caused by them (5-point Likert scale from “not at all” to “very strongly”), their duration (at least 6 months: yes/no), and contextual factors (only related to other physical or psychological problems: yes/no; only related to relationship problem or distressing life situations: yes/no). Higher scores signify more pronounced difficulties or distress. Its psychometric properties have not been tested, yet (see Hoyer et al., 2015).

#### Structure (Axis IV)

The OPD Structure Questionnaire (OPD-SQ) assesses self-reported psychic structural integration on eight scales with 2-3 subscales, respectively (self-perception: self-reflection, affect differentiation, identity; object perception: self/object differentiation, whole object perception, realistic object perception; self-regulation: impulse control, affect tolerance, self-worth regulation; regulation of object relationship: balancing of interests, anticipation; internal communication: experiencing affects, bodily self, use of fantasies; communication with the external world: making contact, communication of affect, empathy; attachment capacities with regard to internal objects: internalization, use of introjects; attachment capacities with regard to external objects: accepting help, severing attachments). Ninety-five items are rated on a 5-point Likert scale from 0 (“completely false”) to 4 (“completely true”). Higher scores represent stronger limitation of the structural capacities. Its validity and reliability have been confirmed (Zimmermann et al., 2010; Ehrenthal et al., 2012; Dinger et al., 2014; König et al., 2016; Jauk and Ehrenthal, 2020).

#### Conflicts (Axis III)

The OPD Conflict Questionnaire (OPD-CQ) is a 66-item self-report instrument measuring six conflicts (individuation versus dependency, submission versus control, need for care versus self-sufficiency, self-worth conflict, guilt conflict, Oedipal conflict) in an active and passive mode of processing, as well as impaired perception of conflicts and affects due to defense on a 5-point Likert scale from 0 (“completely false”) to 4 (“completely true”). Higher scores stand for a more distinct presence of conflicts. OPD-CQ has shown links to measures of structural integration, psychological distress, and life satisfaction; and to add predictive value to structural measures when predicting the latter two. Internal consistencies for most scales were good, apart from four scales which showed insufficient α-values below 70 (see Benecke et al., 2018).

### Analysis

Central to the analysis were the OPD conflict and structure questionnaires; remaining measures served for sample characterization. From two control samples of “healthy” individuals (the inclusion criterion were no experiences with psychotherapy) collected in previous validation studies of the OPD-CQ (Benecke et al., 2018) and OPD-SQ (Ehrenthal et al., 2012) we created matching samples of the same size based on age, education, and relationship status (relationship status only being available for OPD-SQ data). Sample characteristics are summarized in Table 2. Matching was conducted with R software (version 3.6.2, package MatchIt). We compared samples using paired-samples *t*-tests and Wilcoxon tests when data were not normally distributed. Effect sizes were calculated using Pearson's *r* and interpreted in accordance with Cohen (1988, 1992) as *r* < 0.30 representing a small, *r* > 0.30 a medium, and *r* > 0.50 a large effect.

**Table 2:**
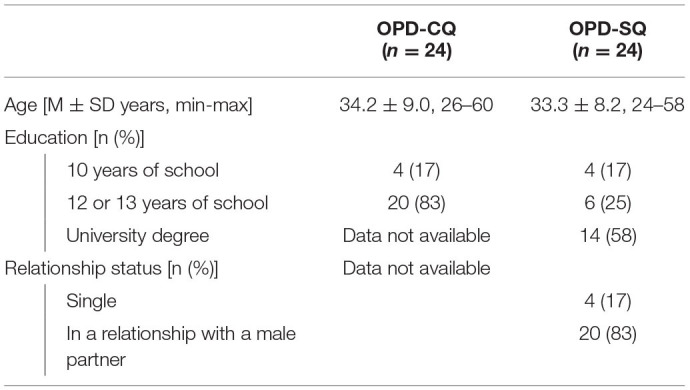
Sample characteristics control samples.

*Percentages rounded; M, mean; SD, standard deviation*.

## Results

### Psychological Problems and Quality of Life

Participants from the clinical sample reported interference from a range of psychological states in the past week. Comparatively high means (> 2 as center of the scale) were found for overly worrying (*M* = 2.4, *SD* = 1.3) and feelings of interpersonal sensitivity (*M* = 2.1, *SD* = 1.2). The average total score was 13.4 (*SD* = 6.3).

Concerning their quality of life, participants from the clinical sample reported moderate psychological health (55.7%, *SD* = 17.3) and quality of social relationships (53.8%, *SD* = 20.4) on average, and slightly better physical health (69.0%, *SD* = 19.2). Satisfaction with environmental factors was rated highest (73.4%, *SD* = 16.0).

### Sexual Difficulties

Next to sexual pain complaints, 15 participants from the clinical sample also perceived distress from lack of sexual interest for at least 6 months in the past year, and 7 participants suffered from problems with orgasm over the same time period. Those last 7 participants fulfilled criteria for clinical relevance as to all three types of sexual difficulties; they also reported high sexual pain frequency and distress caused by it.

With regard to the four weeks prior to participation in the survey, participants from the clinical sample obtained lowest average scores for pain and vaginismus over all domains (for pain: *M* = 2.3, *SD* = 2.0; for vaginismus: *M* = 2.4, *SD* = 2.3). Sexual desire was on average rated as similarly low (*M* = 2.7, *SD* = 1.3). Arousal, lubrication, orgasm, and satisfaction reached moderate mean scores (between 3.3 and 3.8), implying that difficulties were occasionally present but not very pronounced. Participants reached and average total score of 19.2 (*SD* = 8.5), and of 21.6 (*SD* = 9.9) when including the additional vaginismus item.

### Structure

Tables 3 and 4 show results of the comparison of OPD-SQ scales between our clinical participants and the matched control sample. Mean or median differences reached a *p*-value of 0.01 or below for the subscales self-reflection (*p* = 0.002, *r* = 0.59), affect differentiation (*p* = 0.007, *r* = 0.53), and the total score of self-perception (*p* = 0.002, *r* = 0.58); impulse control (*p* = 0.007, *r* = 0.53), self-worth regulation (*p* = 0.008, *r* = 0.52), and the total score of self-regulation (*p* = 0.004, *r* = 0.56); experiencing affect (*p* = 0.009, *r* = 0.53) and bodily self (*p* = 0.008, *r* = 0.54); and the total score of OPD-SQ (*p* = 0.007, *r* = 0.52). The mean difference for the total score of internal communication attained a *p*-value of 0.001 (*r* = 0.63). For all those scales, mean or median values of the clinical sample were higher than values of the control sample.

**Table 3:** Comparison of OPD-SQ scores (paired-samples *t*-test).

**OPD-SQ Scales**	**M (Case)**	**SD (Case)**	**M (Control)**	**SD (Control)**	**t**	**df**	***p***
**Cognitive ability: self-perception**							
Self-reflection	1.67	1.02	0.91	0.63	−3.48	23	0.002
Affect differentiation	2.04	1.11	1.10	0.99	−2.97	23	0.007
Total	1.71	0.96	0.92	0.71	−3.40	23	0.002
**Cognitive ability: object perception**							
Self/object differentiation	1.77	0.56	1.35	0.63	−2.51	23	0.020
Whole object perception	1.49	0.78	1.24	0.78	−1.19	23	0.245
Realistic object perception	1.42	0.66	1.44	0.82	0.06	23	0.952
Total	1.56	0.55	1.34	0.60	−1.35	23	0.192
**Capacity for regulation: self-regulation**							
Impulse control	1.78	1.00	1.06	0.62	−2.96	23	0.007
Affect tolerance	1.64	1.13	0.91	0.62	−2.58	23	0.017
Self-worth regulation	2.07	0.99	1.44	0.76	−2.91	23	0.008
Total	1.83	0.85	1.14	0.53	−3.23	23	0.004
**Capacity for regulation: regulation of object relationship**							
Balancing of interests	1.41	0.83	1.07	0.71	−1.44	23	0.163
Anticipation	1.60	0.83	1.17	0.59	−2.43	23	0.023
Total	1.51	0.73	1.12	0.61	−2.09	23	0.048
**Emotional ability: internal communication**							
Use of fantasies	2.22	0.87	1.74	0.93	−2.13	23	0.045
Total	1.73	0.72	1.09	0.62	−3.93	23	0.001
**Emotional ability: communication with the external world**							
Making contact	1.66	1.14	1.15	1.09	−1.75	23	0.093
Communication of affect	1.53	0.88	1.01	0.62	−2.45	23	0.022
Empathy	1.69	0.91	2.09	1.03	1.40	23	0.176
Total	1.63	0.69	1.42	0.72	−1.12	23	0.274
**Attachment capacity: internal objects**							
Internalization	1.50	0.95	1.23	0.81	−1.10	23	0.284
Use of introjects	2.03	0.84	1.33	0.98	−2.63	23	0.015
Total	1.77	0.80	1.28	0.81	−2.10	23	0.047
**Attachment capacity: external objects**							
Accepting help	1.86	0.95	1.66	1.16	−0.61	23	0.551
Severing attachments	2.39	1.19	1.73	0.95	−2.29	23	0.032
Total	2.13	0.75	1.69	0.85	−1.85	23	0.077
Structure Total	1.73	0.61	1.25	0.57	−2.95	23	0.007

*M, mean; SD, standard deviation; t, t-value; df, degrees of freedom; p, sig. value*.

**Table 4:** Comparison of OPD-SQ scores (wilcoxon test).

**OPD-SQ Scales**	**Median**	**Median**	**T**	**z**	***p***
	**(Case)**	**(Control)**			
Cognitive ability:					
self-perception					
Identity	1.38	0.63	168.5	−2.38	0.017
Emotional ability:					
internal communication					
Experiencing affects	1.25	0.75	240.5	−2.60	0.009
Bodily self	1.13	0.38	191.5	−2.65	0.008

*T, test statistic (sum of positive ranks); z, z score; p, sig. value*.

### Conflicts

Results of the comparison of OPD-CQ scales between the clinical and the matched sample are presented in Tables 5 and 6. A *p*-value of 0.01 or below was obtained by mean or median differences of the active form of the guilt conflict (*p* = 0.004, *r* = 0.60), the passive form of the Oedipal conflict (*p* = 0.009, *r* = 0.51), and the active form of individuation versus dependency (*p* = 0.01, *r* = 0.52). Mean or median differences of the passive form of the guilt conflict (*p* < 0.001, *r* = 0.70), the passive form of the self-worth conflict (*p* = 0.001, *r* = 0.70), and the total score of the passive mode (*p* < 0.001, *r* = 0.68) reached a *p*-value ≤ 0.001. Only for the active form of the guilt conflict scale, the mean value attained by the control sample was higher than the one of our clinical sample; the opposite was the case for mean or median values of all remaining scales mentioned. This signifies a stronger presence of most of the conflicts (except for the active form of the guilt conflict) in the clinical sample.

**Table 5:** Comparison of OPD-CQ scores (paired-samples *t-*test).

**OPD-CQ Scales**	**M (Case)**	**SD (Case)**	**M (Control)**	**SD (Control)**	**t**	**df**	***p***
Impaired perception of conflicts & affects (defense)	1.69	0.60	1.61	0.61	−0.42	23	0.680
Need for care versus self-sufficiency active	2.10	0.60	2.07	0.71	−0.28	23	0.785
Need for care versus self-sufficiency passive	2.21	0.80	1.96	0.70	−1.12	23	0.273
Self-worth conflict active	1.35	0.79	1.77	0.60	2.43	22	0.024
Guilt conflict active	1.13	0.49	1.62	0.48	3.24	23	0.004
Guilt conflict passive	2.20	0.97	0.97	0.82	−4.74	23	0.000
Oedipal conflict active	1.39	0.52	1.43	0.56	0.26	22	0.798
Oedipal conflict passive	2.07	0.67	1.52	0.70	−2.85	23	0.009
Active Mode Total	1.53	0.37	1.63	0.25	1.10	23	0.284
Passive Mode Total	1.95	0.62	1.32	0.46	−4.50	23	0.000

*M, mean; SD, standard deviation; t, t-value; df, degrees of freedom; p, sig. value*.

**Table 6:** Comparison of OPD-CQ scores (wilcoxon test).

**OPD-CQ Scales**	**Median (Case)**	**Median (Control)**	**T**	**z**	***p***
Individuation versus dependency active	1.25	0.75	189.0	−2.57	0.010
Individuation versus dependency passive	1.80	1.33	205.5	−2.06	0.040
Submission versus control active	1.79	1.86	127.5	−0.64	0.520
Submission versus control passive	1.88	1.25	194.5	−2.21	0.027
Self-worth conflict passive	1.75	0.50	231.5	−3.42	0.001

*T, test statistic (sum of positive ranks); z, z score; p, sig. value*.

## Discussion

All women whose data were included in the final clinical sample had still experienced continuous sexual pain over the past year and had felt distressed by it. Additionally, more than half of the sample reported persistent and distressing lack of sexual interest, and almost a third showed difficulties with both sexual interest and orgasm in addition to sexual pain. Global psychological distress assessed by SCL-K-9 was comparatively high (on the 95th percentile of normative values for German women, see Petrowski et al., 2019). Overall, mean/median values of most OPD-SQ scales, including the total score, were higher for clinical participants than for controls. But considering our small sample size and the number of tests performed, results need to be interpreted with caution. We therefore evaluated our data from an exploratory perspective, exploring them for the most robust comparative group differences. The scales that reached *p*-values ≤ 0.01 and large effect sizes in the comparison of mean/median values formed a pattern marked by impaired structural capacities regarding the participants themselves, rather than relating to others: Self-perception, self-regulation and internal communication were mostly affected in comparison to the control sample. The comparison of internal communication total scores obtained the largest effect. This speaks for participants from the clinical sample struggling more with allowing for the perception of states or emotions, and with experiencing them in a dynamic and true-to-life manner. Regarding self-perception and internal communication, participants from the clinical sample reported increased problems with affect differentiation and experiencing affects. Difficulties with cognitively understanding and regulating emotions, in clinical settings known as alexithymia (Taylor et al., 1999), have been found to be associated with somatization (Waller and Scheidt, 2006; Bailey and Henry, 2007; Mattila et al., 2008), a process which is assumed to be involved in certain sexual difficulties of psychological origin. Van Lankveld et al. (1996) identified links between somatization and vulvar vestibulitis syndrome (vulvovaginal pain syndrome, i.a. implicating pain during and/or after sexual intercourse). Moreover, Ciocca et al. (2013) rated over 50% of women with vaginismus in their study as alexithymic or alexithymic in trend, compared to 18% in the control group. Internal communication was not only more affected in the clinical sample in terms of experiencing affects, but also with regard to their bodily self. This refers to the ability to view one's own body in a realistic way and to have lively bodily experiences; impairment can manifest for example in ego-dystonic perception of the body as separate from the self (OPD Task Force, 2008). This is in line with the association between somatoform dissociation – the involvement of body functions in dissociative phenomena – and vaginismus, dyspareunia, and orgasmic disorders in women found by Farina et al. (2011).

Furthermore, participants from the clinical sample demonstrated more difficulties with self-regulation than controls in terms of self-worth regulation and impulse control. The higher mean value of self-worth regulation matches previous findings of women with vaginismus showing lower self-esteem than their partner (Kennedy et al., 1995), or women with vulvar vestibulitis syndrome stating stronger fear of negative evaluation from others than a group of women with orgasmic problems (Brotto et al., 2003). Lundqvist and Bergdahl (2005) also concluded that low self-esteem might be influential in depression and anxiety among women with vestibulodynia. Impaired impulse control could be interpreted as conforming with difficulties with self-perception and internal communication, especially given that respective OPD-SQ items mainly refer to anger. Considering clinical participants' limited capacities of self-reflection, it is plausible that this constellation of structural difficulties results in outbursts of frustration as emotional needs cannot be identified, and can therefore likely not be met either.

Concerning the interpretation of OPD-CQ data, we applied the same exploratory strategy. In comparison to the control sample, participants from the clinical sample obtained higher mean/median values (with the comparative test reaching *p*-values ≤ 0.01 and large effects) for the passive forms of the guilt conflict, self-worth conflict, Oedipal conflict, and the total score of the passive mode, as well as for the active form of the individuation versus dependency conflict. Largest effects were found for the passive forms of the guilt and self-worth conflict. Only the active form of the guilt conflict was more pronounced in the control than in the clinical sample, which does not counter the result that participants from the clinical sample generally leaned more toward its passive form. In total, the conflicts that appeared to be more relevant for our clinical sample, together with the propensity for the passive mode, involve the perceived inferiority and invalidation of the self, and a tendency toward internalizing rather than seeking interpersonal conflict. This matches the pattern of affected structural capacities, which likewise showed more problems with experiencing and regulating oneself than interpersonal relationships. The orientation toward the self is also inherent to the active form of the individuation versus dependency conflict, in which interpersonal closeness is avoided and attachment needs are suppressed in favor of an exaggerated display of autonomy. Whereas a self-worth conflict is more concerned with the valence of the self, the guilt conflict more with feelings of responsibility, and the Oedipal conflict with feeling admirable, attractive and able to compete with others, all their passive forms involve a degradation of the self of some sort.

Since sexual difficulties are determined by a multitude of factors such as relationship dynamics, social aspects, physiological changes (e.g., ageing), and conditions affecting physical health (e.g., endometriosis, vulvovaginal infections, injuries through childbirth), thorough diagnostics are indispensable for appropriate treatment recommendations. For vaginismus and dyspareunia in which by definition a physiological cause is ruled out, our psychodynamically oriented data offer new insights for clinical practice. They suggest that psychotherapeutic approaches may benefit from attending to more general tendencies for self-invalidation and internalization as well as to difficulties with self-perception and potential alexithymia. Especially the results with regard to experiencing affects and the bodily self emphasize the added value of therapeutic strategies involving the body for this type of complaints, as for example Sensate Focus exercises (see Avery-Clark et al., 2019; Hauch, 2019), since patients/clients might struggle with verbal access to their experiences.

The analysis presented here is preliminary, as there are several limitations to our study. Our sample was small, which is not unusual considering that we gathered data from a clinical sample, and of a condition often associated with shame. Previous studies on women with sexual pain difficulties have been based on comparative sample sizes (e.g., de Jong et al., 2009; Borg et al., 2011; Dewitte et al., 2017). Furthermore, even though our matched control samples were assumed to be “healthy controls” based on previous experience with psychotherapy, it is not confirmed that none of them were experiencing recurrent or persistent sexual difficulties at the time of data collection. Nevertheless, given that we recruited a sample that mainly consisted of former psychotherapy patient, a sample of women who had not taken the step of entering such treatment is an acceptable start for comparison.

It is important to note that even though the concepts of psychodynamic conflicts and structure involve assumptions about their development, and the occurrence of certain conflicts and structural deficits in our participants suggests that these developmental assumptions may hold for sexual pain and difficulties with intercourse as well, our data do not allow for an etiological interpretation as they stem from a cross-sectional study. Sexual difficulties might have just as well-influenced the formation of the conflictual and structural pattern we found. For this reason our results have to be treated as indication for potential risk factors for or associations with dyspareunia and vaginismus, whose relationship with the sexual symptoms is yet to be determined.

In summary, our study provides a first approach using psychodynamic assessment according to OPD-2 to investigate the experience of persistent and distressing painful or infeasible intercourse in women. To our knowledge, there is no empirical research on psychodynamic conflicts or structure in women with vaginismus and dyspareunia available, yet. Future research needs to explore whether the same pattern of conflicts and personality functioning can be found in a larger sample of these women, and among different clinical groups experiencing for example other forms of sexual difficulties or psychosomatic conditions. Particularly fruitful would also be an examination of whether women whose sexual complaints would have formerly been diagnosed as vaginismus show similarities or differences in their psychodynamic profile compared to women who would have been diagnosed with dyspareunia. Insight into this question would not only provide information on the development of these conditions, which then would be beneficial to psychotherapy, but would also test the plausibility of recent diagnostic changes.

## Data Availability Statement

The dataset presented in this article is not readily available because the dataset involves data from former patients of the Institute for Sex Research, Sexual Medicine, and Forensic Psychiatry, and is therefore not available for public access. Requests to access the datasets should be directed to t.koops@uke.de.

## Ethics Statement

The study presented in this article was reviewed and approved by Ethical review board of the Hamburg Chamber of Psychotherapists. The patients/participants provided their written informed consent to participate in this study.

## Author Contributions

TK and PB: conceptualization, resources, and funding acquisition. TK, CW, JE, and PB: methodology, writing – review, and editing. TK and CW: software, formal analysis, and data curation. TK, JE, and PB: validation. TK: investigation, writing – original draft preparation, visualization, and project administration. PB: supervision. All authors contributed to the article and approved the submitted version.

## Conflict of Interest

The authors declare that the research was conducted in the absence of any commercial or financial relationships that could be construed as a potential conflict of interest.
